# Physical exertion and working efficiency of reforestation workers

**DOI:** 10.1186/1745-6673-6-20

**Published:** 2011-06-28

**Authors:** Alastair NH Hodges, Michael D Kennedy

**Affiliations:** 1 Faculty of Physical Education and Recreation, University of Alberta, Edmonton, AB, T6G 2H9, Canada

## Abstract

**Background:**

The purpose of this study was to quantify the physical exertion during tree planting work and to examine the relationships between exertion, task efficiency, and productivity.

**Methods:**

Heart rate (HR) was monitored on 34 tree planters while they worked. HR data was collected for a complete working day on 19 subjects and for shorter periods of time on 15 subjects. Video of work tasks was recorded on 22 subjects (video was recorded on 7 of the subjects for whom HR was monitored through a full working day) and analyzed for working pace and proportion of time spent on each task.

**Results:**

HR during a full day (9.0 ± 1.2 hours) of tree planting work was 115.2 ± 8.8 beats.min^-1^, and working HR was 128.2 ± 15.6 beats.min^-1 ^for 82.5 ± 6.8% of the work day. Mean work pace was 452 ± 174 trees.h^-1^, and the proportion of time spent planting each tree was 53 ± 8% of the working time. Significant (P < 0.05) positive correlations were found between work pace and experience level, and between work pace and working HR, and a significant (P < 0.05) negative correlation was found between experience level and HR for a given work pace. No significant relationships were found between experience level or work pace and the proportion of time spent planting each tree.

**Conclusions:**

Tree planters work at approximately 65% of age-predicted HR_max_, and maintain HR at approximately 59% of HR_max _throughout the entire working day. Productivity in these workers appears to be related to effort rather than to experience or task efficiency *per se*.

## Background

Harvesting of Canada's forests, which cover almost 250 million hectares of land and account for 27% of the country's land mass [1], is an important industry and vital part of the Canadian economy, worth approximately $6 billion per annum [2]. In the province of British Columbia in the 2008/09 fiscal year, manual tree planters reforested approximately 230 million tree seedlings over an area of 180,000 hectares [3]. Tree planting is typically piece work (work paid according to unit production - in this case on a per tree seedling basis) involving heavy lifting and walking over significant distances each day, and remote worksites with relatively primitive living conditions for the workers. For these reasons it is often assumed that this work is physically and psychologically taxing, but relatively little data is available on the physical nature of this occupation or on the characteristics of this seasonal worker population. Several studies published over 15 years ago investigated cardiovascular and muscular strain [4], work stress [5,6], and ergonomic aspects [7] of tree planting in Canadian tree planters. It was found that these workers carried loads of approximately 17 kg for distances of 16 km per day with an average heart rate (HR) of 60 - 70% of maximal heart rate (HR_max_) [4,5,7], and had elevated serum levels of creatine kinase, lactate dehydrogense, and aspartate transaminase during the working season [4].

However, there has been a significant change in the silviculture industry (the reforestation and tending of new forests on an industrial basis) in Canada in the past two decades that has affected the physical nature of tree planting and the productivity of the workers, potentially making a significant difference to the occupational characteristics since the previous research was conducted. Specifically, in the mid-1990s, there was a dramatic change in most foresters' philosophy of microsite selection (the process of selecting the best precise location) for each tree seedling that eliminated the need for screefing (removal of forest litter and fermenting humus layers) thereby dramatically speeding up the process of planting each tree and causing significant changes in the mechanics of the work. As a result, the physical nature of the work has changed significantly in the two recent decades leading to a paucity of valid recent data on the nature of this occupation. Some recent work has examined body composition changes [8], and physiological and biochemical stress [9] in these workers, leading to the generalized conclusions that tree planting is an occupation requiring high levels of physical exertion represented by working at 60 - 75% of HR_max _for 57% of the working day [9], and long-term negative energy balance leading to weight loss [8]. However, an examination of the productivity level, the physical exertion, and the efficiency of these workers has not been undertaken.

In the forestry industry, piece work has been identified as a factor which increases injury rates and affects effort [10]. Based on the current incentive model of piece work, Toupin et al. [11] propose that, in silviculture brush cutting workers, pace of work influences effort (as measured by HR) which in turn influences productivity. This model of incentive-based work productivity may be similar amongst other piece rate silviculture workers such as tree planters, although this relationship has not been specifically explored in tree planters. In addition, it is a common anecdotal observation that productivity increases with experience level in these workers, but to date there has been no examination of the relationship of productivity to experience level, nor of the potential mechanism by which the relationship may change with increasing experience. Therefore the purpose of this study was to describe physical exertion (by HR response) during reforestation work, to measure productivity in workers of varying experience level, to analyze the efficiency of tree planters through video analysis, and to examine the relationships between productivity, exertion, and efficiency. It was hypothesized that work pace would be related to experience level, that more experienced tree planters would have lower HR for a given work pace and would spend a smaller proportion of time planting each tree seedling than less experienced tree planters. It was also hypothesized that work pace would be positively related to HR.

## Methods

### Subjects

Data collection occurred at two remote work sites located in north-western British Columbia (within 70 km of the town of Houston) and north-eastern Alberta (within 40 km of the town of Robb), Canada. Data collection occurred in the middle of a reforestation season (June and July) to avoid making measures on workers who were still learning tasks. Twenty males and 14 females participated in this study. The subjects had a mean experience of 3.4 ± 2.3 seasons, with three of the subjects being in their first season of reforestation work. Age, height, and weight was self-reported by subjects as precise measurement was not necessary for this study. Written informed consent was obtained from all subjects before participation as approved by the Faculties of Physical Education and Recreation, Agricultural, Life and Environmental Sciences, and Native Studies Research Ethics Board at the University of Alberta.

### Experimental Protocol

Heart rate was recorded on 19 subjects while they completed a full day (9.0 ± 1.2 hours) of tree planting work, and on 15 subjects during shorter periods (1.7 ± 1.2 hours) of work. Video of 22 of the subjects was recorded while they worked. Video was recorded on 7 of the 19 subjects for whom HR was measured during a complete day and all 15 of the other subjects. Therefore all analyses involving measures from video recording were made on 22 subjects. Measurements of HR and video recording were time matched such that the HR for a given segment of work analyzed by video could be subsequently identified.

### Heart Rate Assessment

Subjects were instrumented with a memory equipped HR monitor (Suunto Memory Belt). HR was recorded every 10 seconds until the recording period was complete and the data were downloaded from the HR monitors to a computer using specialized software (Suunto Training Manager version 2.3.0.15). All HR data were exported as text files for subsequent analysis in Microsoft Excel (Microsoft Corporation Office Professional Edition 2003).

### Video-recording & Analysis

Subjects were video-recorded on a digital camcorder (Sanyo Xacti) during three minutes of tree planting work. Though subjects had agreed to have their work filmed, they were unaware of the time point they were being filmed in an attempt to avoid a possible Hawthorne effect. Each subject was filmed on three separate occasions to ensure consistency of the data captured by video (retest correlation coefficient, r = 0.93). All video data were downloaded as Moving Picture Experts Group 4 (MP4) files to a computer and a time-analysis was completed on each file using video viewing software (Windows Media Player version 6.4.09.1130, Microsoft Corporation). This analysis was performed manually by a researcher with 16 years of tree planting experience by recording the total time spent planting each tree, the total number of trees planted, and the total time spent walking between each tree. Time spent planting each tree was defined as the time between the point at which the worker stopped walking and had clearly selected a microsite and the point at which they closed the earth around the tree seedling and began walking again.

### Data & Statistical Analyses

Mean HR for a full working day was calculated as the average HR throughout the entire work day (including breaks in work) on 19 subjects. Working HR was defined as the mean HR recorded during the process of planting trees (as opposed to mean HR inclusive of all activities during the work day), and was calculated by including only the data recorded during the three minutes of video recording. Work pace was defined as the number of trees planted per hour. This was calculated by summing the number of trees planted in the three minute video sample of work to the nearest tree and multiplying by 20. Heart rate per work pace was calculated by dividing working HR for each subject by the work pace they exhibited at that time point in the work day. The proportion of time spent planting trees versus time spent walking between each tree was calculated. Efficiency was defined as the quotient of HR divided by the working pace, with a lower quotient indicating a higher efficiency. In order to calculate the proportion of the total working day that was spent at the working HR (rather than on breaks), a graph of HR vs. working time was generated for each subject and then visually analyzed to assess the data points that corresponded with working times and resting times. Break periods were defined by a drop in HR below the average working HR for that individual that extended into several minutes of time. (An example of the visual pattern is clear on Figure 2). The total working time was summed for each individual subject.

Pearson product-moment correlations were calculated to analyze the relationships between working HR and work pace, work pace and experience level, and HR / work pace (efficiency) and experience level, with statistical significance set at α = 0.05 for all calculations. Reliability of the video analysis for work pace was performed by correlation analysis between two sets of different video on each subject.

## Results

**Figure 1:**
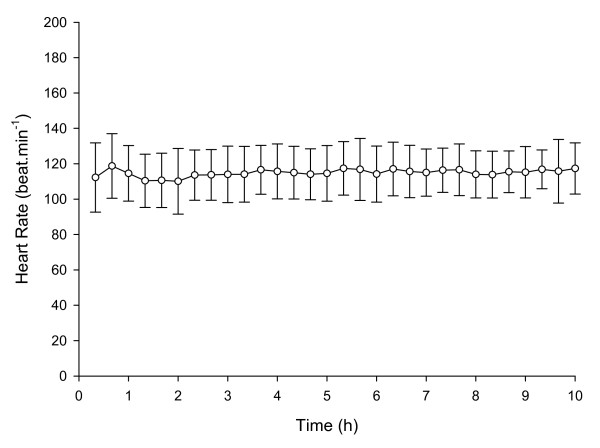
**Mean heart rate**. Mean heart rate during 10 hours of tree planting work (n = 19).

**Figure 2:**
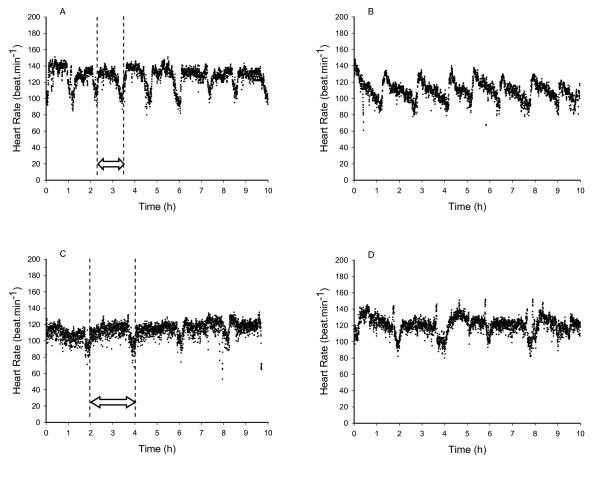
**Sample heart rates**. Sample individual heart rate responses during 10 hours of tree planting work. Panels A & B: experienced tree planters (4 and 5 seasons experience respectively). Panels C & D: inexperienced tree planters (both during first season of work). Dashed lines on panels A & C denote one work period or "bag-up" for comparison purposes.

**Figure 3:**
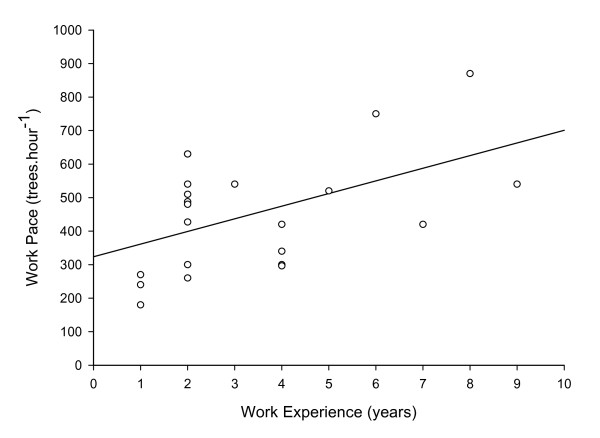
**Work pace and worker experience**. Work pace versus worker experience, r = 0.50, P < 0.05 (n = 22).

**Figure 4:**
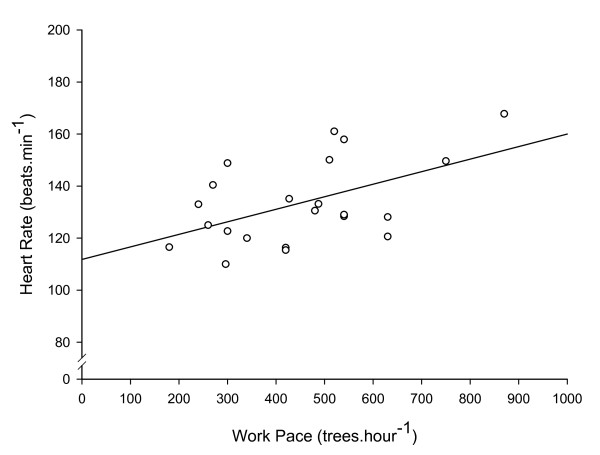
**Heart rate and work pace**. Heart rate versus work pace, r = 0.52, P < 0.05 (n = 22).

**Figure 5:**
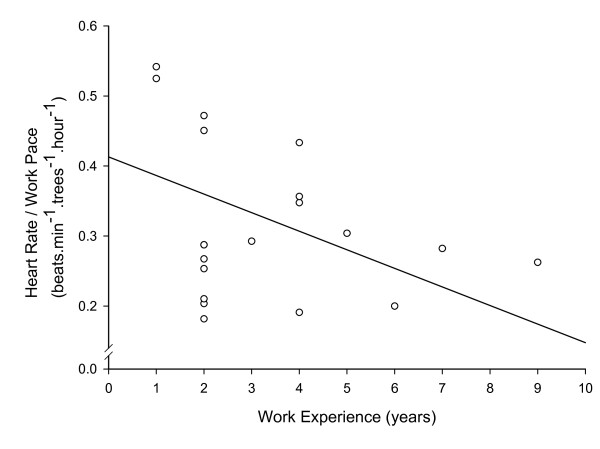
**Efficiency and worker experience**. Heart rate / work pace versus worker experience, r = -0.47, P < 0.05 (n = 22).

Subjects were 23.9 ± 3.9 years of age, 174.5 ± 10.2 cm in height, and had a mass of 68.4 ± 12.2 kg. Mean HR during 9 hours of tree planting work (including breaks in work), was 115 ± 9 beats.min^-1 ^(Figure 1). Mean working HR during tree planting work was 128 ± 16 beats.min^-1^. For those subjects on whom full day HR data was collected, the proportion of time during each day spent at the working HR ranged from 71 - 94% of the work day (mean 83 ± 7%), with the individual HR response of each subject found to be cyclical throughout the working day (Figure 2). Mean work pace was 452 ± 174 trees.hour^-1^, and the mean proportion of working time spent planting each tree was 53 ± 8% versus 47 ± 8% spent walking between each tree. A significant (P < 0.05) positive correlation of r = 0.50 was found between tree planting experience level and work pace (Figure 3), a significant (P < 0.05) positive correlation of r = 0.52 was found between work pace and working HR (Figure 4), and a significant (P < 0.05) negative correlation of r = - 0.47 was found between work experience and HR / work pace (Figure 5). No significant relationship was found between the proportion of time spent planting each tree and experience level, or the proportion of time spent planting each tree and work pace. Intra-rater reliability of the video analysis of work pace was r = 0.93.

## Discussion

Manual reforestation (tree planting) in Canada is seasonal work often thought of as physically taxing [12]. Wide variation in productivity between workers has been anecdotally observed for decades amongst this population and, since tree planting is almost invariably piece work involving pay rates based on production, there is a large benefit to both workers and employers to increased productivity [11]. However, the nature of the individual differences in productivity, and the potential mechanisms for these differences have not previously been investigated in this population.

### Main Findings

Tree planters sustain an average HR of approximately 115 beats.min^-1 ^(59% of age-predicted HR_max _[13]) throughout a full work day, with working HR of approximately 128 beats.min^-1 ^(65% of age-predicted HR_max _[13]) during approximately 83% of the full day, with a cyclical HR response throughout the work day indicative of the nature of the work which includes periods of planting trees followed by rest periods required to re-load with tree seedlings. Mean working pace was 452 ± 173 trees.h^-1^, and the proportion of time spent planting each tree was 53 ± 8% of total working time. A significant negative correlation was found between experience level and HR for a given work pace, and a significant positive correlation was found between experience level and work pace, and between work pace and working HR. No significant relationship was found between experience level and the proportion of time spent planting each tree.

### Effort as determined by heart rate response

The separation of exertion, as measured by HR, into mean daily HR of 115 beats.min^-1 ^and mean working HR of 128 beats.min^-1 ^is novel to this study, though the mean physiological response to the work is similar to that previously reported [4,5,9]. The cyclical nature of the HR response to tree planting work, and the individual variation in this response observed in this study is also relatively novel. Banister et al. [5] previously reported a similar cyclical pattern in the HR response to tree planting, but with much longer cycle periods and a much greater differentiation between experienced and inexperienced workers than that found in the current study. This finding may be related to the previously described changes in the industry in the past two decades, reflecting changes in the exertion and work to rest patterns of these workers. Tree planters use canvas or soft synthetic buckets called planting bags strapped around their waist to carry tree seedlings. Depending on seedling size and personal preference, workers typically carry 200 - 600 tree seedlings at a time before needing to reload. In this industry each load is referred to as a "bag-up", with multiple bag-ups during a work day being interspersed with short breaks to reload with tree seedlings. This work - rest cycle is evident in the cyclical HR response to a full day of work found in this study. Figure 2 shows several individual examples of the variation in this cyclical response throughout the workday. There is noticeable variation in this pattern between experienced versus inexperienced workers. The main differences are less well defined HR differences between work and rest periods, and fewer work intervals (bag-ups) in a given time period (5 vs. 8 bag-ups in 10 hours) for the inexperienced worker. Figure 2 clearly illustrates that the experienced workers complete their bag-ups in approximately half the time of the inexperienced workers. Therefore, the experienced workers are more productive as determined by Toupin [11], although an exact number of tree seedlings planted per bag-up was not recorded. It can therefore be inferred that experienced tree planters work faster compared to inexperienced tree planters and the experienced workers don't require a greater working HR to achieve this greater productivity. In addition to the HR pattern over a working day as demonstrated in Figure 2, the average work pace as calculated from video analysis showed that the experienced workers' pace was more than double that of the inexperienced workers (487 ± 160 vs. 230 ± 46 trees.h^-1^). Unfortunately the sample size of inexperienced workers was too small (n = 3) to group the subjects into purely inexperienced vs. experienced workers with any statistical confidence. However, the significant positive correlation between experience level and work pace (r = 0.50, P < 0.05) confirms our hypothesis that productivity increases with experience in these workers. To our knowledge, this is the first study that confirms the anecdotally held view that working pace increases with experience level in these workers.

The mean working heart rates were similar to previous findings in this population [4,5,9], despite significant change in industry standards and tree planting technique since some of this earlier work. This may support the notion that these workers exert themselves to a given maximal sustainable effort regardless of the technical difficulty of the task. This maximal sustainable effort has been defined as critical power in a sporting context where human performance at or below critical power can be maintained indefinitely [14]. In an industry in which earnings vary directly with production, it is likely that workers find the maximal daily exertion level that is both tolerable and maintainable, and strive to achieve this level regardless of the precise nature of this work. If this is the case, the working HR of 128 beats.min^-1 ^and daily average HR of 115 beats.min^-1 ^likely represent this maximal maintainable exertion level. This could be further investigated by examining the HR response to tree planting on terrains of varying difficulty. Productivity is anecdotally known to vary significantly in these workers depending on the difficulty of the terrain (typical production may vary as widely as a minimum of 1,000 trees per day on difficult terrain to over 4,000 on easy terrain). Although this relationship has yet to be explored, it appears from the findings of this study that physical exertion, as measured by HR, has not changed despite a reduction in the amount of work required to plant each tree and a significant increase in worker productivity in the past two decades.

### Exertion and efficiency

The negative correlation found between tree planting experience level and HR for a given work pace confirms the hypothesis that more experienced tree planters have lower HR for a given work pace. Since efficiency was defined as a lower HR for a given work pace, this denotes a positive relationship between experience level and efficiency. However, our hypothesis that more experienced tree planters would spend a smaller proportion of time per tree than less experienced tree planters was rejected. Therefore, our findings indicate that more experienced workers are both more productive and efficient at their work, but we failed to isolate the mechanism for this greater efficiency. The positive correlation found between work pace and HR confirms the hypothesis that work pace is related to effort as described by HR, and may indicate that the increased productivity of more experienced workers is simply a result of greater physical effort.

Tree planting work is relatively technical in nature and appears to require a high degree of skill. Both the mechanics of planting trees and the process of microsite selection improve with experience, leading to a much greater planting pace (and therefore earnings). It was surprising, however, that there was no relationship observed between experience level and the proportion of time spent planting each tree. The mean proportion of time spent planting trees was 53 ± 8% compared to 47 ± 8% spent between each tree, and these ratios did not vary with experience level. This is an important finding for this population, and can be interpreted in one of two ways. Firstly, it could be concluded that the rate at which a worker plants a tree in comparison to their rate of microsite selection (i.e. time spent walking between trees) does not affect productivity. Alternatively, and more plausibly in our opinion, it could be concluded that more experienced tree planters have increased the rate at which they perform both tasks equally: that experienced workers are equally faster at the mechanics of planting each tree and at the skill of microsite selection between each tree in comparison to less experienced workers, and that improvements in productivity therefore require skill in each of these tasks. Therefore we speculate that more experienced workers are able to support a higher physical effort (as described by higher HR) due to their greater skill at the tasks involved, thereby allowing them to set a higher pace, whereas less experienced workers are forced to set a lower pace due to a lower skill at both the tasks of planting each tree seedling and of walking between sites while searching for a suitable microsite.

Contrary to the hypothesis of this study was the finding that working HR was higher in more experienced workers. We found quite a strong relationship indicating that more experienced workers simply exert themselves more than their less experienced co-workers. Indeed, the finding that more experienced workers were more efficient (defined as a lower HR for a given planting pace), only strengthens the statement that more experienced tree planters exert themselves more than less experienced tree planters. In order to achieve the higher working HR with a higher working efficiency, the more experienced tree planters' working pace had to be greater than that reflected only by the higher working HR.

Giguere and colleagues [7] have previously analyzed the working time as ranging between 55 - 61% of the total work day. Though the present study did not directly record such a breakdown of work times through observation and recording of times, it was calculated quite accurately from the pattern of heart rate responses in each individual as described in the methods. From this observation, the time spent planting trees ranged from 71 - 94% of the work day (83 ± 7%), which is significantly higher than previously reported [7]. This indicates that, compared to their counterparts almost two decades earlier, modern tree-planters spend significantly more time planting trees during their working day. Since the prior study was published in 1993, the only plausible explanation for this difference is a change within the industry in the intervening years.

## Conclusions

Tree planting work involves significant physical exertion, demonstrated by an average daily HR of 115 beats.min^-1 ^and an average working HR of 128 beats.min^-1 ^over a period of 10 hours. Though more experienced tree planters appear to be more efficient, as observed by the lower HR per working pace, the mechanism of their efficiency is not well explained by this study. Specifically, there was no relationship between the proportion of time spent planting each tree and the experience level of the workers. In addition, more experienced workers were more productive than their less experienced counterparts, and this increased productivity appears to be a combination of greater efficiency and greater physical exertion. Ultimately, the findings of this study indicate that productivity amongst manual tree-planters is achieved through increased exertion and increased efficiency, rather than through alterations in the mechanics of the work. The findings indicates that there are perhaps ways in which working efficiency increases with experience level, though surprisingly it does not appear linked to the ability of the workers to alter the proportion of time spent planting each tree or proportion of time spent in microsite selection.

## Competing interests

The authors declare that they have no competing interests.

## Authors' contributions

AH designed the study, carried out the data collection, participated in the data analysis, and drafted the manuscript. MK participated in the analysis and drafted the manuscript. All authors read and approved the final manuscript.
